# Carbonized Cow Dung as a High Performance and Low Cost Anode Material for Bioelectrochemical Systems

**DOI:** 10.3389/fmicb.2018.02760

**Published:** 2018-11-30

**Authors:** Huajun Feng, Zhipeng Ge, Wei Chen, Jing Wang, Dongsheng Shen, Yufeng Jia, Hua Qiao, Xianbin Ying, Xueqin Zhang, Meizhen Wang

**Affiliations:** ^1^Zhejiang Provincial Key Laboratory of Solid Waste Treatment and Recycling, School of Environmental Science and Engineering, Zhejiang Gongshang University, Hangzhou, China; ^2^Zhejiang Lantu Environmental Protection Co., Ltd., Hangzhou, China; ^3^Department of Military Installations, Army Logistics University of PLA, Chongqing, China; ^4^Advanced Water Management Centre, The University of Queensland, Brisbane, QLD, Australia

**Keywords:** bioelectrochemical system, cow dung, carbonization, anode material, current generation

## Abstract

We develop a high-performance anode formed from carbonized cow dung for bioelectrochemical systems. Thermal gravimetric analysis showed that the CD carbonization process started at 300°C and ended at approximately 550°C; the weight was reduced by 51%. After a heat-treatment at 800°C for 2 h, the treated CD featured a good conductivity and a high specific surface area. The maximum current density of 11.74 ± 0.41 A m^-2^ was achieved by CD anode (heated at 800°C), which remained relatively stable from more than 10 days. This study shows that a valuable anode material can be produced through conversion of CD by high-temperature carbonization. This approach provides a new way to alleviate environmental problems associated with CD.

## Introduction

Bioelectrochemical systems (BESs) are bioreactors designed to use microbes as catalysts to extract energy directly from organics in wastewater (Logan and Rabaey, 2012). Currently, practical applications of this technology are limited by its low current densities and high costs (Feng et al., 2016; Liang et al., 2016). The electrodes, the key compositions of BESs, primarily determine the current output and capital cost of BESs. Improving the specific surface area and biocompatibility of electrodes enhance bacterial adsorption and growth, which improve the current output performance. Among the wide range of electrode materials, carbon-based electrodes, such as carbon cloth, carbon felt, and graphite plates, are extensively used in microbial fuel cells (MFCs) because of their excellent biocompatibility and chemical stability. However, one of the greatest drawbacks to large scale applications is the prohibitive cost of these materials (50–150 US m^-2^), which contributes to more than half of the entire reactor costs (Guo et al., 2015; Feng et al., 2016). Therefore, it is necessary to develop alternative high-specific surface area and cost-effective electrodes for BESs.

Looking for cost effectiveness and performance affinity of electrode materials catalyzed the attempts to apply biochar in MFCs. Biochar is the product of heating biomass in the absence of oxygen and this carbonized material was used to be applied as soil amendment to fix nutrition or adsorbent to remove pollutants (Biswas and Mishra, 2016). Recently, its properties of high conductivity and high porosity were verified and it was regarded as a feasible carbon material for electrode fabrication (Cai et al., 2016). Moreover, the wide feedstocks (such as corn stem, king mushroom and sawdust) of biochar production, biochar is easily accessible and cost effective, making it to be promising for electrode configuration in the practical application of MFCs (Karthikeyan et al., 2015).

Today there are an estimated 1.4 billion cows around the world, which produce approximately 4.2 million tons of cow dung (CD) per year (Reddy et al., 2014; Yusef et al., 2017). To address this issue and recycle CD, various means have been implemented, such as traditional composting to produce fertilizer (Fan et al., 2006) and modern anaerobic digestion to extract renewable biogas (Yokoyama et al., 2007). Recently, the conversion of CD into biochar has arisen as another effective means for treating CD. CD is rich in organic compounds including protein, hemicellulose, and cellulose and other available forms of nitrogen (Yusef et al., 2017); hence, a CD-derived biochar is expected to contain an abundance of nitrogen- and oxygen-functional groups, which might promote higher current production than other feedstocks current production in BESs.

In this study, we show that valuable anode materials can be produced through conversion of CD by high-temperature carbonization. We also investigated the BES the current generation performances of these anodes.

## Experimental

### Preparation of Cow Dung Electrodes

Dry cow dung (CD) (Xiulin Fertilizer Industry Co., Ltd.) was purchased as precursor to prepare the electrode material. The CD was mixed with certain proportion of water (g/g = 3/4), packed into a mold (2 cm × 1.3 cm × 0.5 cm), and then dried at room temperature (rectangle in shape with an actual area of 2.1 cm^2^ and thickness of 0.4 cm). The molded CD pieces were carbonized in a tube furnace for 2 h at different temperatures of 400, 600, 800, and 1000°C under a N_2_ flow rate of 300 mL min^-1^. After the carbonization was completed, a piece of titanium wire was bonded to the carbonized waste mass with conductive epoxy to ensure an electrical connection. The CD materials, subjected to the different carbonization temperatures of 400, 600, 800, and 1000°C, are denoted as CD400, CD600, CD800, and CD1000, respectively.

### Characterization

The surface morphology of the carbonized CD was characterized with a scanning electron microscope [SEM, G (pro), Phenom, Holland]. X-ray photoelectron spectroscopy (XPS) was used to measure the elemental compositions. The high resolution O1s (528–546 eV) and C1s (281–300 eV) peaks were measured on an EscaLab 250Xi spectrometer with a monochromatic Al Kα source (Thermo, United Kingdom). Fourier Transform infrared spectroscopy (FTIR; Bruker, VERTEX 70, Germany) was used to detect the surface functional groups on the electrode samples. Thermogravimetric analysis (TGA) measurements were performed on a TA Q600 thermogravimetry/differential scanning calorimetry (TG/DSC) instrument to measure the weight loss over the temperature range 0–1200°C in a nitrogen atmosphere. Specific surface areas of the CD-based electrodes were measured through Quadrasorb SI (Quantachrome Instruments, America). The biofilms developed on electrodes were subjected to the LIVE/DEAD BacLight^TM^ bacterial viability tests (LIVE/DEAD1 BacLight Bacterial Viability Kit, Molecular Probes, America) following the manufacturer’s instructions. Labeled cells were visualized and z-stacks were captured on a Zeiss LSM 780 confocal laser scanning microscope (CLSM). Microbial protein on the electrode was obtained using an ultrasonic cell disrupter (JY92-IIN, China) and high-speed freezing centrifuge (D-37520 Osterode, Germany). The amount of biological protein was determined by an ultraviolet spectrophotometer (UV-2600, Japan) according to the Coomassie brilliant blue methods.

### Electrochemical Measurements

All the electrochemical tests were performed in a three-electrode system using a potentiostat (Biologic, France). The CD-based electrodes were used as the working electrode, with a graphite plate electrode as the auxiliary electrode, and an Ag/AgCl reference electrode in 50 mL sterile M9 solution. Electrochemical impedance spectroscopy (EIS) was measured at open circuit potential with an amplitude of 10 mV over a frequency range of 100 kHz to 100 mHz. The Nyquist plots were fitted with a suitable equivalent electrical circuit (by fitting of the equation (R1 + R2/Q2 + R3/Q3, where R1 is ohmic resistance, R2 is charge transfer resistance, and R3 is diffusion resistance of the anode, respectively).

### MFC Start-Up and Operation

An MFC reactor, as described by Guo et al. (2014) was constructed with eight working electrodes (WE, CD electrodes), sharing the same counter electrode (CE, carbon felt) and reference electrode (RE, Ag/AgCl 3 M KCl). The anode chamber of the reactor was filled with 500 mL of M9 solution (NH_4_Cl, 0.1 g L^-1^; NaCl, 0.5 g L^-1^; KH_2_PO_4_, 4.4 g L^-1^; K_2_HPO_4_, 3.4 g L^-1^; MgSO_4_, 0.1 g L^-1^, NaHCO_3_, 2 g L^-1^), trace elements (FeSO_4_ 7H_2_O, 1.0 mg L^-1^, CuSO_4_ 5H_2_O, 0.02 mg L^-1^, H_3_BO_3_, 0.014 mg L^-1^, MnSO_4_ 4H_2_O, 0.10 mg L^-1^, ZnSO_4_ 7H_2_O, 0.10 mg L^-1^, Na_2_MoO_4_ 2H_2_O, 0.02 mg L^-1^, CoCl_2_ 6H_2_O, 0.02 mg L^-1^), and 1 g L^-1^ of sodium acetate as an electron donor (Liu et al., 2004; Feng et al., 2016). For the reactor start-up, 50 mL of anodic effluent from an available acetate-fed MFC reactor that had been continuously running in our lab for 2 years was inoculated into the anodic compartment. The catholyte solution was M9 solution. The anodes were controlled at a potential of -0.2 V vs. Ag/AgCl by a potentiostat (Biologic, France) and current data were collected by chronoamperometry (CA). The acclimation experiments of all the reactors were performed in batch mode at 30°C.

## Results and Discussion

### Morphology of the Electrodes

**FIGURE 1:**
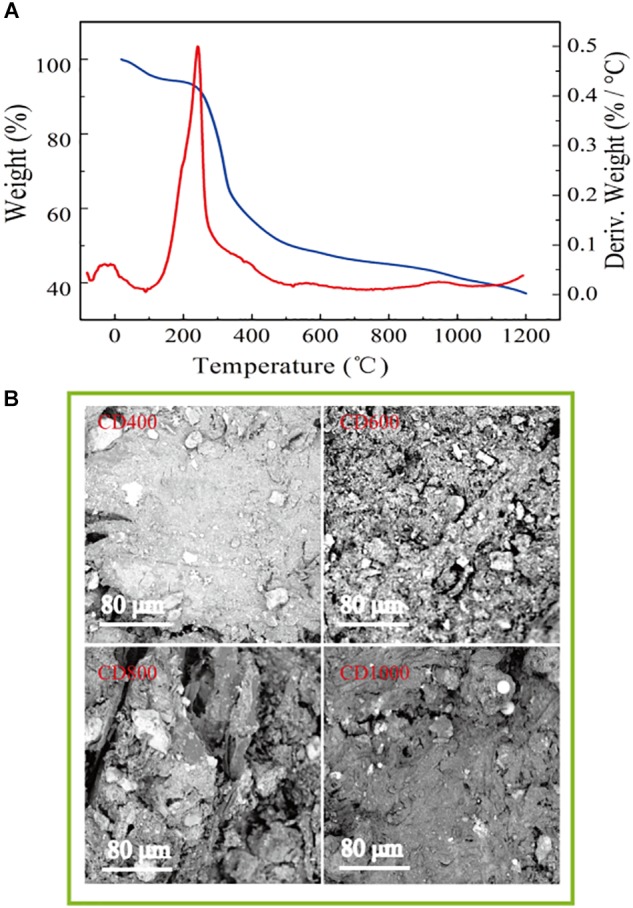
**(A)** TG/DSC plots of waste tire raw materials (blue: TG, red: DSC) **(B)** The image of SEM of the CD400, CD600, CD800, and CD1000.

The results of TG/DSC indicated that the CD carbonized process started at 300°C and the material was reduced by 51% when it halted at about 550°C, caused by cracking of the organic matter. The weight continued to decline more gradually as the temperature was further increased (Figure 1A).

**FIGURE 2:**
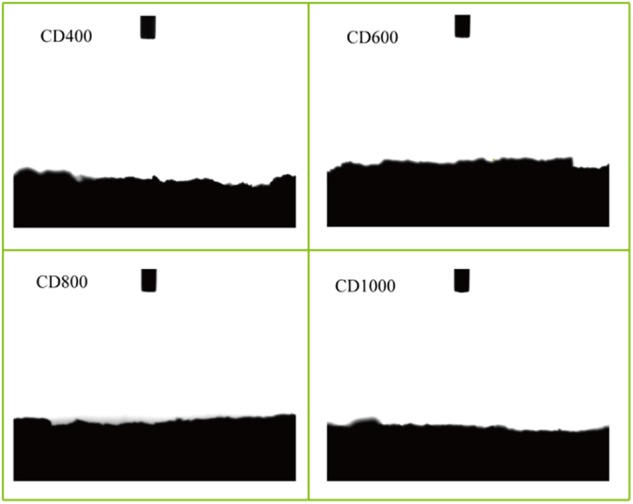
Water contact end illustration of CD electrode after different carbonization temperature.

SEM images also showed that the surface of the CD (heated at 600, 800, and 1000°C) was porous and the pore size of carbons particles was approximately several tens of micrometers, which is likely beneficial for microbial adhesion. Among the samples that have been carbonized at different temperatures, the surface of CD800 appeared to be the roughest. The volume of CD1000 decreased slightly after the carbonization and the pore sizes in the carbon particles were smaller than those of CD800 (Figure 1B). Conversely, the surface of CD400 was relatively smooth because the organic matter was not carbonized effectively. This result was verified by Brunauer–Emmett–Teller (BET) results. The CD800 sample featured the greatest specific surface area of 62.70 m^2^ g^-1^, which was 173 times higher than that of untreated cow dung (0.36 m^2^ g^-1^), followed by CD1000 and CD600, at 62.18 m^2^ g^-1^ and 54.30 m^2^ g^-1^, respectively. The specific surface area of CD400 was relatively low at 1.34 m^2^ g^-1^. All electrode surfaces after carbonization showed completely hydrophilic surfaces with contact angles of 0° (Figure 2). This property should allow bacteria to easily adhere to the surfaces (Liang et al., 2017). Therefore, the CD electrodes heated at 600, 800, and 1000°C showed features benefit bacterial growth.

### Chemical Characteristics of the Electrodes

**FIGURE 3:**
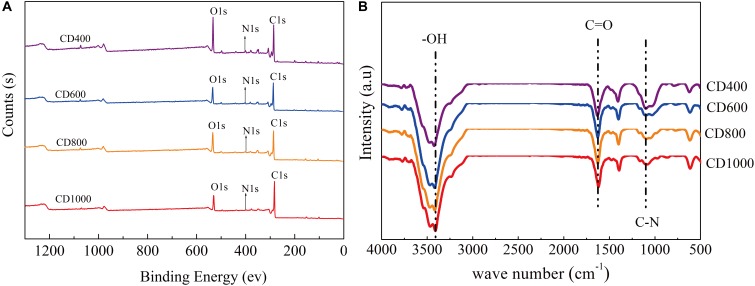
**(A)** XPS spectra of the samples of CD400, CD600, CD800, and CD1000 **(B)** FT-IR of the samples after carbonization at different temperatures of the cow dung.

As shown by the XPS results (Figure 3A), all the CD anodes featured three main peaks at 284, 400, and 532 eV. These were associated with C1s, N1s, and O1s signals, respectively (Gao et al., 2017), arising from the organic matter in the cow dung. High-resolution scans in the N region and corresponding peak fitting indicated that the N signal was composed of three component peaks with corresponding binding energies of 398.5 eV for pyridinic-N, 400.1 eV for pyrrolic/pyridone-N, and 401.2 eV for quaternary-N (Supplementary Figure S1) (Gao et al., 2017). It has been reported that the presence of pyridinic-N, pyrrolic/pyridone-N, quaternary-N in biochar can promote effective electron transfer (Tan et al., 2017). Moreover, quaternary-N in the carbon matrix and bonded with three carbon atoms can also improve the conductivity of carbonaceous materials (Hong et al., 2014; Gao et al., 2017; Tan et al., 2017). We found that the quantity of associated quaternary-N were 25% (CD400), 46% (CD600), 54% (CD800), 63% (CD1000), respectively. Therefore, the CD electrode materials heated at higher temperatures will likely improved electron transfer between bacteria and the anode.

The high-resolution O1s spectra could also be divided into three component peaks, assigned to C = O species at 531.4 eV, O-C = O at 532.4 eV, and C-O-C 533.15 eV (Supplementary Figure S2) (Hong et al., 2014). Groups such as C = O have good electron accepting properties and C-O-C groups are capable of donating electrons. Therefore, these groups can function as redox mediators on the CD surface to accelerate electron transfer (Cardenas-Robles et al., 2013; Dong et al., 2013).

To further study the chemical functional groups of the different CD anodes, FTIR analysis was conducted (Figure 3B). The pristine CD materials featured a broad band at approximately 3434 cm^-1^, which is assigned to the stretching vibration of the hydroxyl groups (Gao et al., 2017). This peak remained after carbonization at different temperatures, indicating the potential for hydrophilic surface properties in the CD anode materials. Peaks at 1632, and 1120 cm^-1^ were assigned to vibrations of C = O and C-N, respectively (Hong et al., 2014; Gao et al., 2017), suggesting the presence of amide groups in the carbonated CD. Previous studies have shown that amide groups can benefit electron transfer from microbes to the anode surface (Ma et al., 2016). Therefore, the existence of -HO, C = O and C-N groups might be beneficial for the use of carbonized CD as a biological anode.

### Electrochemical Characteristics of the Electrodes

**FIGURE 4:**
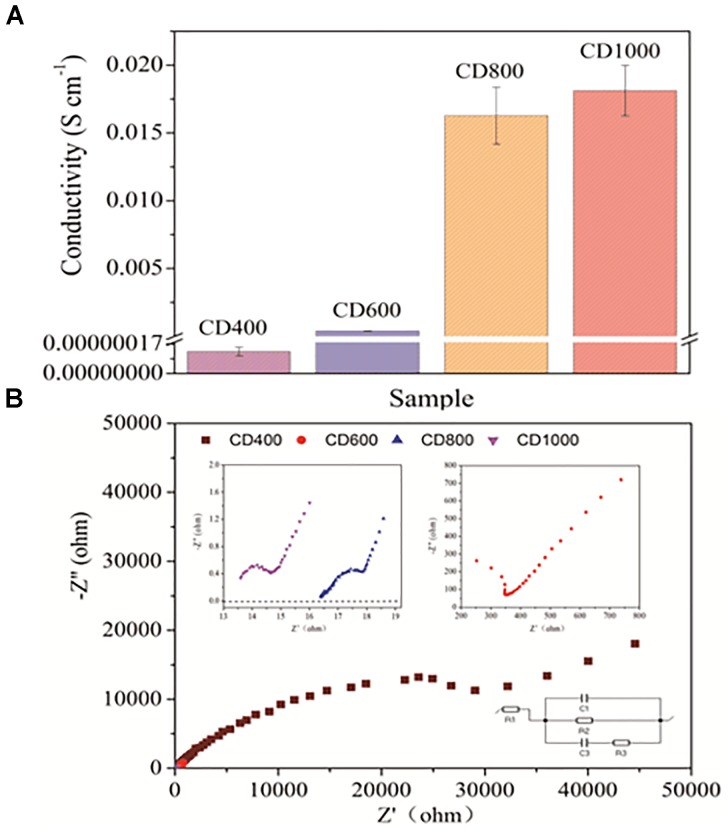
**(A)** Conductivity for CD400, CD600, CD800, and CD1000 **(B)** Electrochemical impedance spectra of the CD anodes at open circuit potential with an amplitude of 10 mV and a frequency range of 100 kHz to 100 mHz in fresh medium.

As shown in Figure 4A, the conductivity performance of the CD was negligible if the CD was not completely carbonized (i.e., heated at temperatures below 550°C as determined from the TG/DSC curves); the CD became conductive (CD600: 0.00039 ± 0.00002 S cm^-1^) when the temperature was increased to 600°C. The conductivity also improved with the increase of carbonization temperature. Notably, the conductivity remained stable when the carbonization temperature was greater than 800°C (CD800: 0.016 ± 0.003 S cm^-1^, CD1000: 0.018 ± 0.002 S cm^-1^). EIS was measured and then fitted by the equation R_1_ + R_2_/Q_2_ + R_3_/Q_3_, where R_1_, R_2_ and R_3_ represent ohmic resistance, charge transfer resistance, and diffusion resistance of the anode, respectively (Figure 4B) (Du et al., 2014). All values of the equivalent circuit parameters could be seen in Supplementary Table S1. As shown in the EIS curves (Figure 4B), the R_2_ value of CD600 was determined to be 662.7 Ω, while those of CD800 and CD1000 were considerably lower, at 1.7 and 1.4 Ω, respectively. This decrease of the charge transfer resistance could be caused by the highly conductive contact area between the biofilm and CD anode surface (Ying et al., 2017). These results indicate that higher temperature for heating treatments resulted in better electrical conductivity. Compared with previous reports of Zheng et al. (2015), the electrode resistance of a CB (carbon black)-binder/SSM (stainless steel mesh) (>40 Ω) was similar to that of the waste in our study. Therefore, the carbonized CD shows suitable electrical characteristics for use as an electrode material.

### Bioelectrocatalytic Current Generation

**FIGURE 5:**
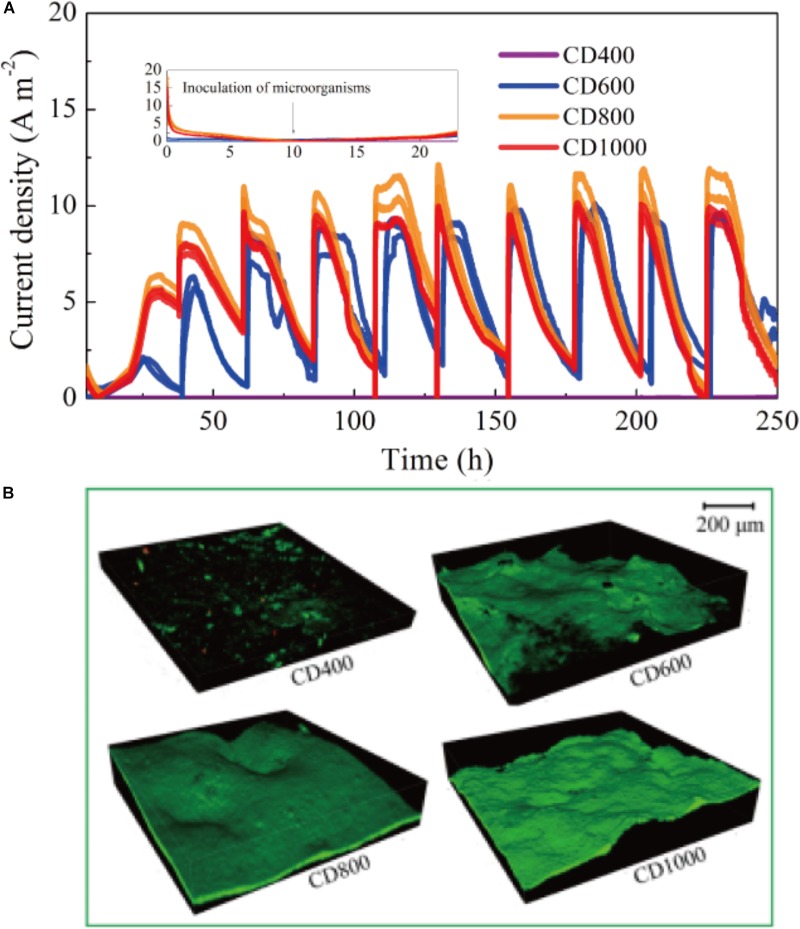
**(A)** Current output over time of BESs with different anodes **(B)** CLSM images of biofilms on CD400, CD600, CD800, and CD1000 electrodes, respectively. Green and red colors represent the live and dead bacteria, respectively.

As shown in Figure 5A all the CD anodes showed current generation after bacteria were added within approximately 10 h. The maximum current density stabilized in the range from 10.03 to 12.15 A m^-2^ after approximately 50 h of operation. To our knowledge, it has been reported that carbon-based anodes are hardly able to generate current and reach a stable maximum current in such a short time, including activated carbon fiber felt (10 days startup time) (Liu et al., 2014), and carbon mesh anode (3–6 days startup time) (Zheng et al., 2015). Among our CD anodes (CD600, CD800, and CD1000), the maximum current density of 11.74 ± 0.41 A m^-2^ was achieved by CD800, which remained relatively stable from more than 10 days.

**FIGURE 6:**
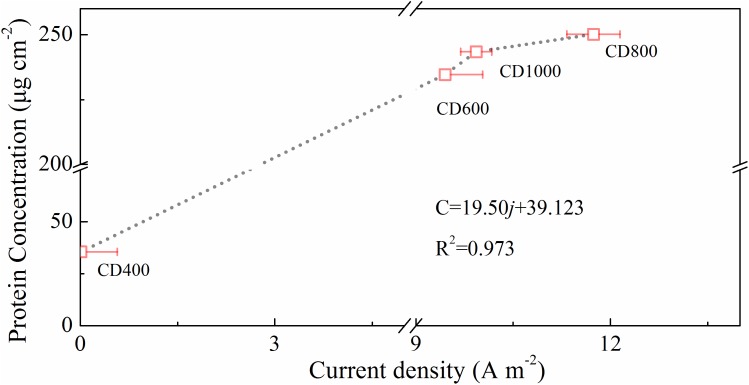
Linear relationship between current density and protein content for CD400, CD600, CD800, and CD1000.

In addition, a thick biofilm covered the whole surface of the CD electrodes (CD600, CD800, and CD1000) and could be seen in CLSM images (Figure 5B). However, almost no biofilm was observed on the surface of CD400, indicating that the non-carbonized CD (i.e., CD treated at temperatures below 550°C) could not be used as bio-anode. The biofilm on the surface of CD800 was slightly thicker (∼50 μm) compared with that of the other samples, which is likely because of the favorable electrode structure (pore size) (Guo et al., 2014). Additionally, the CLSM image of bare CD electrode was almost dark (Supplementary Figure S3). Figure 6 shows a positive correlation between the maximum current output and the microorganism coverage of these electrodes. In addition, live bacteria biofilm (green) covered almost 100% of the specific surface area of the CDs electrodes (CD600, CD800, and CD1000). Therefore, our findings demonstrate that carbonized CD is a promising anode material with high biocompatibility and good stability for BES.

### Economic Evaluation of Electrode Material

Notably, the current density achieved for our CD electrodes was higher than that of previously reported MFCs based on commercial carbon materials (Supplementary Table S2). The current density of carbon felt (carbon mesh) has been reported to be 5.6 A m^-2^ (∼5 A m^-2^), which is half that of our CD anode system. Moreover, the current output performance of our CD anodes also considerably outperformed anodes based on other carbonized biomass, such as tubular bamboo (5.00 A m^-2^), loofah sponge (1.90 A m^-2^), textile (8 A m^-2^) (Zhang et al., 2014; Karthikeyan et al., 2015; Wang et al., 2015; Xie et al., 2015). In addition, we roughly estimated the cost in relation to the preparation of the CD electrodes used in this study. The cow dung was locally available with no charges related to its purchase and transportation (Ma et al., 2016). The overall cost depends mainly on the crushing and pyrolysis steps and the total cost was estimated by recording the electricity used in the process. Compared with other high-performance carbon-base electrodes, such as carbon felt (∼20.3 US m^-2^) and graphite (∼26.1 US m^-2^), the price of our CD electrodes was only ∼16.1 US m^-2^ (Supplementary Table S2). These results indicate that carbonized CD could be a cost-effective carbon anode material for MFCs.

## Conclusion

In this study, we have tested the current output performance of an anode formed from carbonized CD treated at different temperatures (400–1000°C) under a nitrogen atmosphere. The results of our TG/DSC analyses showed that the CD carbonization process started at 300°C and stopped at approximately 550°C. Furthermore, for carbonization at temperatures above 600°C the CD electrodes possessed high conductivity. The maximum current output performance (11.74 ± 0.41 A m^-2^) was obtained for CD treated at 800°C. This study proposed carbonized cow dung could be used as a high-performance anode in BESs, which could convert CD waste into an economical and environmentally friendly electrode material.

## Author Contributions

HF, DS, and MW designed the study. HF and ZG wrote the manuscript. HF, ZG, and WC did much of the testing. JW, YJ, HQ, and XY helped with the data analysis. XZ and MW helped to improve the quality of the manuscript. All authors read and approved the final manuscript.

## Conflict of Interest Statement

JW was employed by the company Zhejiang Lantu Environmental Protection Co., Ltd., China. The remaining authors declare that the research was conducted in the absence of any commercial or financial relationships that could be construed as a potential conflict of interest.
