# Supplementary material for: Carbonized Cow Dung as a High Performance and Low Cost Anode Material for Bioelectrochemical Systems
Source: Front Microbiol. 2018 Nov 30;9:2760. doi: 10.3389/fmicb.2018.02760 (PMC6284060; doi:10.3389/fmicb.2018.02760)
Supplement: Supplementary file 1 [file Data_Sheet_1.docx]

**Supplementary materials**

Carbonized cow dung as a high performance and low cost anode material for bioelectrochemical systems

Huajun Feng^a^, Zhipeng Ge^a^, Wei Chen^a^, Jing Wang^b^, Dongsheng Shen^a^, Yufeng Jia^a^, Hua Qiao^c^, Xianbin Ying^a^, Xueqin Zhang^d^, Meizhen Wang^a*^

^a^ Zhejiang Provincial Key Laboratory of Solid Waste Treatment and Recycling, School of Environmental Science and Engineering, Zhejiang Gongshang University, Hangzhou, 310012, China.

^b^ Zhejiang Lantu Environmental Protection Co.,Ltd., China.

^c^ Department of Military Installations, Army logistics University of PLA, Chongqing, 401311, P.R. China

^d^ Advanced Water Management Centre, The University of Queensland, St Lucia, QLD 4072. Australia

* Address: School of Environmental Science and Engineering, Zhejiang Gongshang University, Hangzhou 310012, P. R. China.

Tel.: +86 571 87397126; fax: +86 571 87397126.

E-mail: [wmzyy@163.com](mailto:wmzyy@163.com)


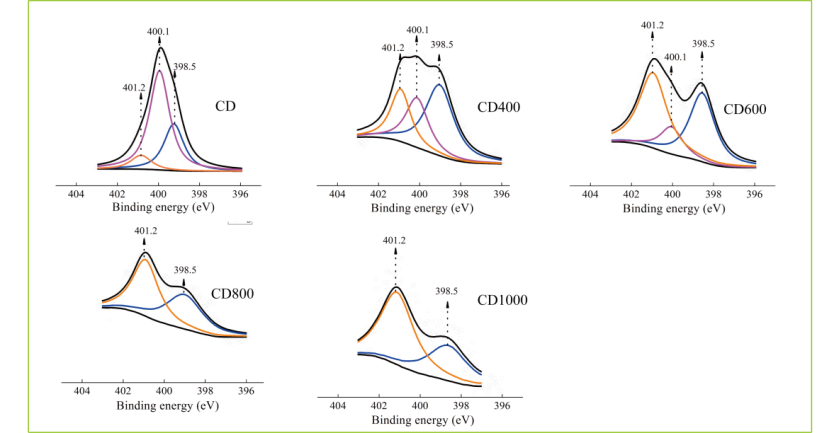


Fig. S1 High resolution N1s spectra and fitting peaks of pristine CD, CD400, CD600, CD800,

CD1000


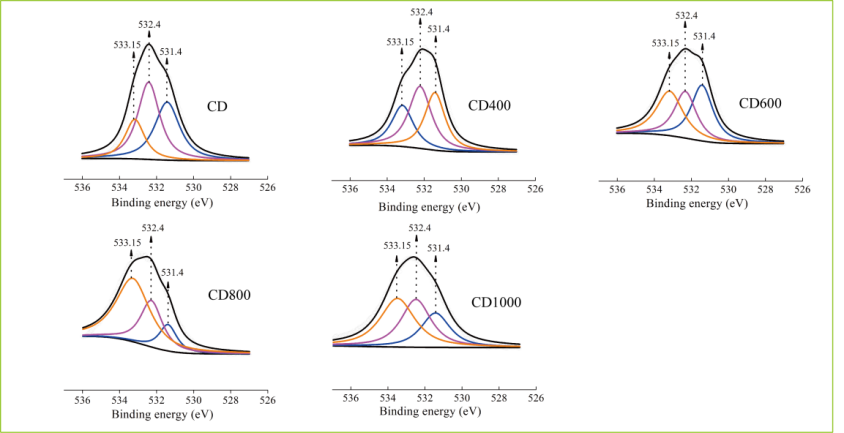


Fig. S2 High resolution O1s spectra and fitting peaks of pristine CD, CD400, CD600, CD800,

CD1000


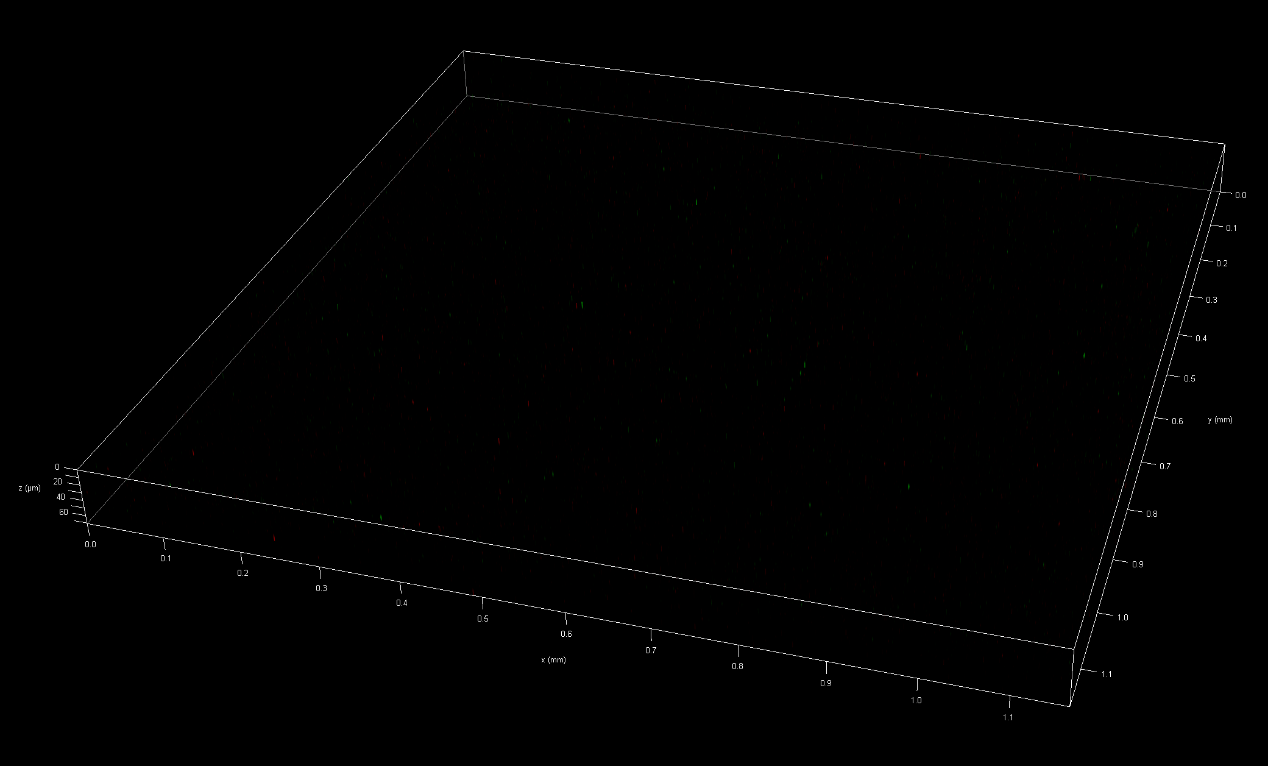


Fig. S3 CLSM images of bare CD electrode (without bacterial).

Table S1 The values of the equivalent electrical circuit (R1+ R2/Q2 +R3/Q3) for CD400, CD600, CD800, and CD1000.

|  | R1 | R2 | R3 |
| --- | --- | --- | --- |
| CD 400 | 198.6 | 10808 | 46422 |
| CD600 | 66.2 | 662.7 | 2425 |
| CD800 | 16.6 | 1.7 | 1592 |
| CD1000 | 13.3 | 1.4 | 557.4 |

Table S2 Examples of commercial carbon and cow dung bioelectrodes invested in BESs

| Bioelectrodes for BESs | Current density(A m^-2^) | Price ($ / m^2^) | Ref. |
| --- | --- | --- | --- |
| Graphite felt | 5.6 | ~20.3 | Feng et al., 2016 |
| Graphite plate | 6.0 | ~26.1 | Liu et al., 2014 |
| Carbon mesh | ~5.0 | - | Xie et al., 2015 |
| Carbon cloth | 0.6 | - | Wang et al., 2015 |
| Cow dung | ~12.15 | ~16.1 | This study |
| ^a^ The total cost of the cow dung electrode was calculated through the recording of electricity. The input of tube furnace for keeping the 800℃ was about 1000 W and the heating time was 3 hours. Based on it, the total heating electricity was about 156.3 kW h / m^2^. In addition, the electricity for crushing was only 4.3 kW h / m^2^. | | | |
